# Anomalies in the refinement of isoleucine

**DOI:** 10.1107/S139900471400087X

**Published:** 2014-03-19

**Authors:** Karen R. M. Berntsen, Gert Vriend

**Affiliations:** a CMBI, Radboud University Medical Center, Geert Grooteplein 26-28, 6525 GA Nijmegen, The Netherlands

**Keywords:** isoleucine, refinement

## Abstract

A study of isoleucines in protein structures solved using X-ray crystallography revealed a series of systematic trends for the two side-chain torsion angles χ_1_ and χ_2_ dependent on the resolution, secondary structure and refinement software used. The average torsion angles for the nine rotamers were similar in high-resolution structures solved using either the *REFMAC*, *CNS* or *PHENIX* software. However, at low resolution these programs often refine towards somewhat different χ_1_ and χ_2_ values. Small systematic differences can be observed between refinement software that uses molecular dynamics-type energy terms (for example *CNS*) and software that does not use these terms (for example *REFMAC*). Detailing the standard torsion angles used in refinement software can improve the refinement of protein structures. The target values in the molecular dynamics-type energy functions can also be improved.

## Abstract 2

The side-chain torsion angles of isoleucines in X-ray protein structures are a function of resolution, secondary structure and refinement software. Detailing the standard torsion angles used in refinement software can improve protein structure refinement.

## 1. Introduction   

In 1991, Engh and Huber published their landmark article on bond lengths and bond angles (Engh & Huber, 1991 ▶). The parameters that they determined are used in nearly all of today’s macromolecular software. Other authors have published parameters for planarities (Hooft, Sander *et al.*, 1996*b* ▶; Sychrovsky *et al.*, 2009 ▶; MacArthur & Thornton, 1996 ▶) and torsion angles (Wang *et al.*, 2008 ▶; Jones & Thirup, 1986 ▶; Clore & Kuszewski, 2002 ▶; Butterfoss *et al.*, 2005 ▶; Hooft *et al.*, 1997 ▶; Ponder & Richards, 1987 ▶; Dunbrack & Cohen, 1997 ▶; Lovell *et al.*, 2000 ▶), while several groups have been working on the use of torsion angles in refinement software (Clore & Kuszewski, 2002 ▶; Berjanskii *et al.*, 2006 ▶; Rice & Brünger, 1994 ▶; Brünger, 1992 ▶; Adams *et al.*, 2010 ▶). The rotamericity of amino-acid side chains has often been studied for purposes such as homology modelling (Wang *et al.*, 2008 ▶), X-ray and NMR refinement (Jones & Thirup, 1986 ▶; Clore & Kuszewski, 2002 ▶), structure validation (Laskowski, MacArthur *et al.*, 1993 ▶; Laskowski. Moss *et al.*, 1993 ▶; Hooft, Vriend *et al.*, 1996 ▶; Lovell *et al.*, 2003 ▶; Read *et al.*, 2011 ▶) or the determination of MD force-field parameters (Lindorff-Larsen *et al.*, 2010 ▶). MacArthur & Thornton (1999 ▶) realised that the average observed values for side-chain torsion angles are resolution-dependent. They concluded that this is caused by the fact that low-occupancy alternate side-chain conformations are often not observable at low resolution, and hypothesized that refinement of a single conformation in density that reflects multiple conformations leads to systematic torsion-angle deviations.

Target values for parameters such as bond lengths and angles are similarly used as data in crystallographic refinement. Consequently, it can also be expected that their final values will be closer to reality at high resolution, when a large amount of X-ray data are available, and closer to the initial target values at low resolution. If these target values can be improved, low-resolution models in particular will improve.

Touw & Vriend (2010 ▶) showed, for example, that the ideal τ angles depend on secondary structure and amino-acid type, and the values observed in PDB files additionally depend on the resolution and the refinement software used. We studied whether or not these factors similarly influence the observed isoleucine side-chain torsion angles. We conclude that determination of the optimal χ_1_ and χ_2_ values is a highly complicated task that the authors of different refinement programs have solved in different ways, all of which are open to improvement. Isoleucine was chosen for a series of reasons: it has two side-chain torsion angles (there are not sufficient data in the PDB to study residues with more variable torsion angles); it is an abundant residue; it is hydrophobic, which means that long-range interactions that are difficult to analyse do not come in play; and it is β-branched, which means that strong inter­actions between the side chain and the backbone are involved in rotamer choice.

## 2. Methods   

All PDB files that have been solved by X-ray diffraction, that contain at least one intact isoleucine and that were released before February 2013 were extracted from the PDBFinder (Hooft, Sander *et al.*, 1996*a* ▶) database and stored in a separate relational database (Touw & Vriend, 2010 ▶). A series of scripts were used to extract the data used in this study. The database and all of the software needed to maintain it are available upon request.

**Figure 1:**
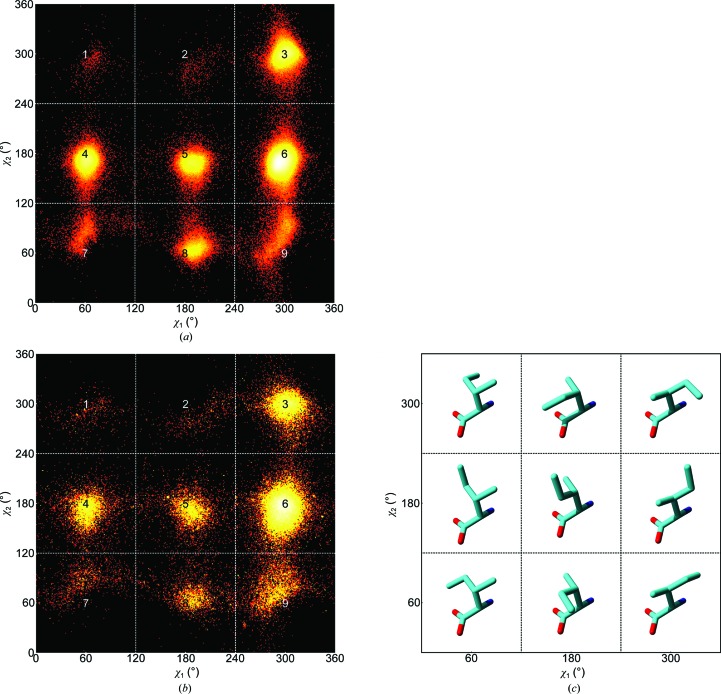
Two-dimensional histograms of the χ_1_ and χ_2_ dihedral angles of isoleucine in structures with resolution ranges of (*a*) 0.0–2.0 Å and (*b*) 3.0–10.0 Å. The data are scaled logarithmically from black (minimum) to white (maximum) and are divided into nine sections of 120 × 120°. The bin size is 1°. A total of 558 287 isoleucines contributed to the high-resolution plot (*a*) and 90 891 contributed to the low-resolution plot (*b*). (*c*) The nine rotamers of isoleucine with χ values of 60.0° (*gauche* ^+^ or g^+^), 180.0° (*trans* or t) or 300.0° (*gauche* ^−^ or g^−^).

**Figure 2:**
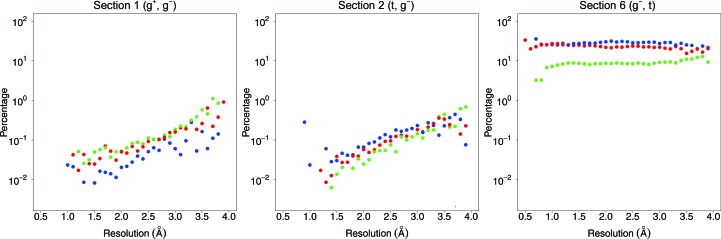
Percentage of isoleucines in three sections as a function of secondary structure and resolution. The sections are as indicated in Fig. 1 ▶. Each dot represents the percentage of all isoleucines in a 0.1 Å wide resolution bin that have a χ_1_–χ_2_ combination corresponding to that section, and the secondary structure as indicated by the colour. Only isoleucines that fulfil the quality criteria of Table 1 ▶ are represented. α-Helix, blue; β-strand, red; loop, green.

Different data-selection protocols were used for different studies. Fig. 6 was based on all isoleucines in the database. Fig. 4 compares today’s data on leucine with the data produced in 1999 by MacArthur and Thornton. For this study, we used the leucines in a *PDB_SELECT* (Hooft, Sander *et al.*, 1996*b* ▶; http://swift.cmbi.ru.nl/gv/select/) data set of 21 667 protein chains that had been solved by X-ray diffraction methods at a resolution better than 3.0 Å and that had an *R* factor lower than 0.25. This data set contains no pairs of sequences that are more than 90% identical upon sequence alignment. Figs. 1, 2, 5, 7(*g*), 7(*h*), 8 and 9 are based on the 1 765 734 isoleucines that fulfilled all of the criteria listed in Table 1 ▶.

The database was augmented with a large number of computationally derived parameters, such as torsion angles, secondary structure, ϕ and ψ, and residual *WHAT_CHECK* (Hooft, Vriend *et al.*, 1996 ▶) quality parameters (summarized in Table 1 ▶). These parameters were obtained using the *WHAT IF* (Vriend, 1990 ▶) web service (Hekkelman *et al.*, 2010 ▶) and *DSSP* (Kabsch & Sander, 1983 ▶; Joosten, te Beek *et al.*, 2011 ▶).

Electron densities from EDS (Kleywegt *et al.*, 2004 ▶) were inspected using *Coot* (Emsley & Cowtan, 2004 ▶) and screenshots were obtained using *PyMOL* (v.1.2.0.1; Schrödinger) and *YASARA* (Krieger *et al.*, 2002 ▶).

Plots were generated using the statistical language R (R Development Core Team, 2012 ▶), employing the plyr (Wickham, 2011 ▶) package for data aggregation, and two-dimensional histograms were generated using Java and *GIMP* (Kimball *et al.*, 1997 ▶).

The *YASARA* (Krieger *et al.*, 2002 ▶) macro for Python (Lutz, 2001 ▶) was used to isolate the first α-helix (Ser6–Cys16) from crambin (PDB entry 3nir; Schmidt *et al.*, 2011 ▶) and to mutate all of its residues to alanines. The hydrogen positions in this α-­helix were determined using the method of Hooft and coworkers (Hooft, Sander *et al.*, 1996*c* ▶; Krieger *et al.*, 2012 ▶). This α-helix was energy-minimized in a simulation cell in vacuum using the NOVA (Krieger *et al.*, 2002 ▶) force field, with nonperiodic boundaries, a 20 Å cutoff for nonbonded interactions and *YASARA*’s Simspeed parameter set to ‘slow’. A short ‘steepest-descent minimization’ was followed by simulated annealing (200 steps of 2 fs; at every tenth step the atom velocities were reduced by 0.9) until the energy improvement was less than 0.05 kJ mol^−1^ per atom. After this minimization, Ala11 was mutated to an isoleucine (χ_1_ = 0, χ_2_ = 0) which was then rotated around its χ_1_ and χ_2_ in steps of 1.0° to obtain 360^2^ different conformations. For each conformation, the total energy (in J mol^−1^) of the system and the atom experiencing the greatest force were stored and used to colour the ln(energy) contours in the χ_1_/χ_2_ energy plots. Atom distance contour plots were produced in a similar way. For this plot, Ile11 was again rotated into 360^2^ different conformations. All methyl and methylene groups were replaced by pseudo-atoms (MB for the C^β^ of alanine and QG, MG and MD for C^γ1^, C^γ2^ and C^δ1^ of isoleucine, respectively) located at their centres of mass. For every conformation, the distances from QG, MG and MD of Ile11 to all other (pseudo) atoms (excluding 1–2 and 1–3 interactions) were calculated. These distances were corrected by the van der Waals radii of the atoms involved (H, 1.20 Å; C, 1.60 Å; N, 1.50 Å; O, 1.45 Å; RM, 2.00 Å; R2Q, 2.00 Å; Tsai, 2007 ▶). The minimum resulting distance per conformation was plotted on a contour plot and the corresponding atom pairs were used as labels to colour this plot. The same two procedures were followed for the β-hairpin Val13–Gln26 from the VP2 subunit of human rhinovirus (PDB entry 1hrv; Rosenwirth *et al.*, 1995 ▶), in which Gly19 (in the β-­turn) was not mutated and Ala23 was mutated back to isoleucine.

Two-dimensional histograms of χ_1_/χ_2_ distributions were fitted against a two-dimensional Gaussian function to determine the local maxima and two-dimensional spreads in each of the nine rotamer sections.

## 3. Results   

The rotamers of isoleucine can be divided into nine sections, which are highly unevenly populated (see Fig. 1 ▶). This is mainly caused by strain owing to interactions between the C^γ^ and C^δ^ atoms of the side chain and atoms in the local backbone. The low population of sections 1 and 2 became even more pronounced when we only analysed high-resolution structures (Fig. 1 ▶ *a*).

Fig. 2 ▶ shows the occurrence of χ_1_–χ_2_ combinations as a function of resolution and secondary structure. At higher resolution, we observe significantly lower populations in sections 1, 2, 7 and 9. Fig. 2 ▶ shows the relative frequencies of isoleucines in sections 1, 2 and 6 as a function of resolution and secondary structure.

**Figure 3:**
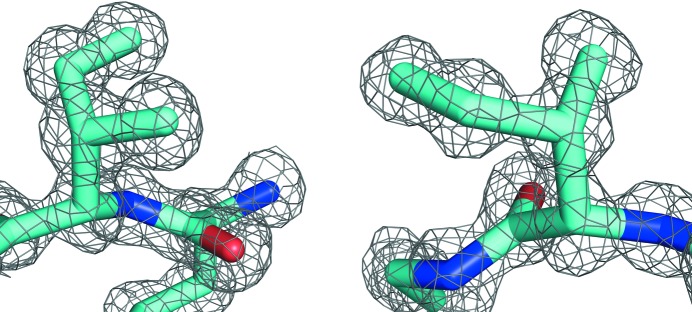
Two examples of rare isoleucine rotamers that were unambiguously observed in good electron density in structures determined at around 1.0 Å resolution. Left: Ile337 in chain *A* of the triacylglycerol lipase protein (PDB entry 1d5t; Brzozowski *et al.*, 2000 ▶) is observed in section 1. Right: Ile15 in chain *B* of the HIV protease (PDB entry 1kzk; Reiling *et al.*, 2002 ▶) is observed in section 2. Electron densities were obtained from the EDS server and are contoured at 1.4σ. At http://swift.cmbi.ru.nl/gv/isoleucine/ the local structures of these residues are shown and the interactions that keep the residues in these unfavourable rotamers are explained. *MolProbity* (Chen *et al.*, 2010 ▶) and *WHAT_CHECK* (Hooft, Vriend *et al.*, 1996 ▶) call these two rotamers ‘poor’. We believe that ‘improbable’ is a better description.

As illustrated in Fig. 2 ▶, there are around ten times fewer isoleucines in sections 1 and 2 at high resolution (∼1.5 Å) than at low resolution (∼3.0 Å). The population differences of sections 1 and 2 (see Figs. 1 ▶ and 2 ▶) suggest that the incidence of isoleucines in these sections could be zero if we look solely at structures solved at extremely high resolution. However, inspection of two examples (see Fig. 3 ▶) reveals that χ_1_–χ_2_ torsion-angle combinations corresponding to sections 1 and 2 do occur in high-resolution protein structures. We compared all isoleucines in the original PDB files with the corresponding isoleucines in the *PDB_REDO* databank (Joosten & Vriend, 2007 ▶; Joosten, Joosten *et al.*, 2011 ▶). We studied 951 631 isoleucines that have all atomic *B* factors less than 80 Å^2^ and all atomic occupancies of 1.0 in both databases. We observe that sections 1 and 2 are less populated in the *PDB_REDO* files. In most cases we also observe that χ_2_ is different. A full list of observed differences (also as a function of secondary structure, in absolute and relative terms) is available from http://swift.cmbi.ru.nl/gv/isoleucine/.

**Figure 4:**
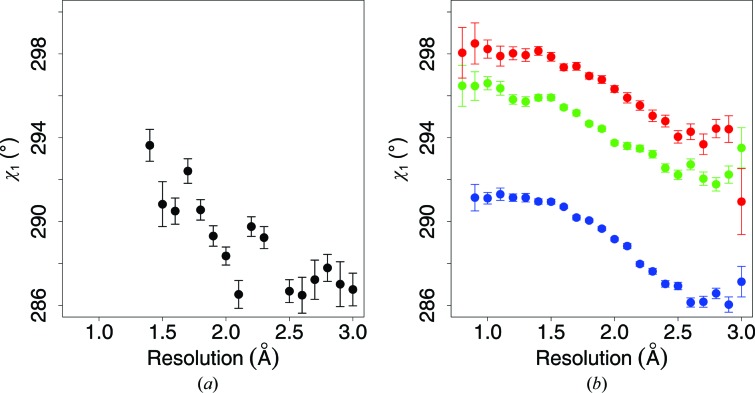
Mean value of the χ_1_ *gauche* ^−^ rotamer as a function of resolution and secondary structure for leucine (independent of χ_2_). (*a*) Values reconstructed from the study of MacArthur & Thornton (1999 ▶). (*b*) As in (*a*) but for a much larger data set (see §[Sec sec2]2) and subdivided for secondary structure. Colour coding is the same as in Fig. 2 ▶. Each dot represents at least 50 observations. Error bars represent the standard errors of the mean.

In 1999, MacArthur and Thornton studied the resolution-dependence of χ_1_ for the 17 relevant amino-acid types (MacArthur & Thornton, 1999 ▶). At the time, they could only study χ_1_ because they did not have sufficient data to study details such as multiple side-chain torsion angles or secondary structure. They did, however, show a detailed plot for the *gauche* ^−^ angles of leucine as a function of resolution. Purely for reference, we compared this plot with the values that we obtained, split up into three secondary-structure types. Fig. 4 ▶ shows the resolution-dependence of χ_1_ in leucine as determined by MacArthur and Thornton in 1999 and by our group in 2013. This plot deals with leucine rather than isoleucine because Mac­Arthur and Thornton presented data for leucine but not for isoleucine. Fig. 4 ▶ confirms the previously observed resolution-dependence of the torsion angles, but it also shows that this dependence is more complicated than the near-linear behaviour observed in 1999.

**Figure 5:**
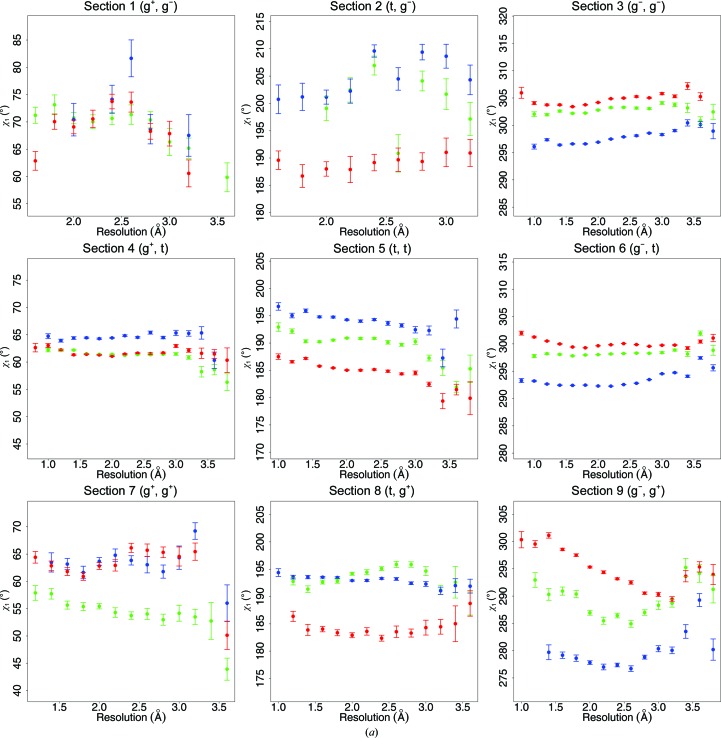

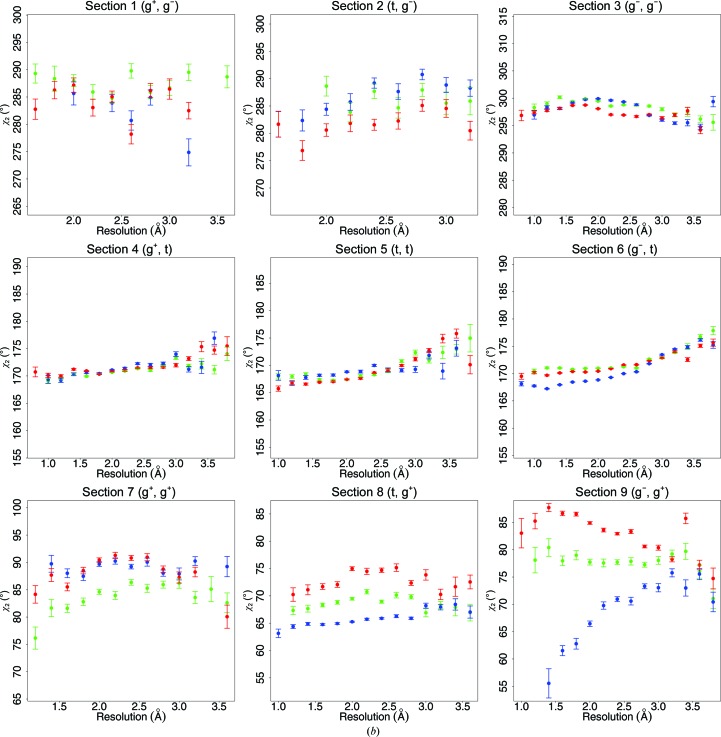
The average side-chain torsion-angle values of Ile as a function of resolution and secondary structure. Each dot represents the mean of at least 50 data points. Error bars represent the standard errors of the mean. The nine panels in (*a*) show χ_1_ values. The nine panels in (*b*) show χ_2_ values. Colours are the same as in Fig. 2 ▶. In both (*a*) and (*b*), the panels are placed in the same order as in Fig. 1 ▶. Consequently, similar torsion angles are oriented vertically in (*a*) and horizontally in (*b*). The resolution bin size is 0.2 Å.

Fig. 5 ▶ shows the average isoleucine side-chain torsion angles in the nine sections of Fig. 1 ▶ as a function of resolution and secondary structure. This figure shows that these values not only differ between different resolutions and secondary structures, but that the resolution dependence also differs between the nine sections. When comparing a single row or column in Fig. 5 ▶, the average value of one χ often depends on the rotamer of the other χ.

It is tempting to look for trends in the 18 panels of Fig. 5 ▶. For example, the mean value of the χ_1_ *gauche* ^−^ rotamer is always largest for β-strand and smallest for α-helix, independent of χ_2_, while the mean value of the χ_1_ *trans* rotamer is always smallest in β-strand. The χ_1_ *gauche* ^+^ rotamer, however, does not show such trends. The average values in a loop often lie between the average values in α-­helix and β-strand, but in five of the 18 panels this is not the case. χ_1_ is more strongly influenced by the secondary structure than χ_2_, as might be expected from the smaller distance between χ_1_ and the backbone compared with the distance between χ_2_ and the backbone. The secondary structure has the least influence on χ_2_ when it adopts a *trans* conformation, corresponding to the C^δ^ atom pointing away from the backbone. As expected, the combination χ_1_, χ_2_ = *gauche* ^+^, *trans* shows the least secondary-structure influence on both χ_1_ and χ_2_. However, the differences in the mean values are only marginally significant.

**Figure 6:**
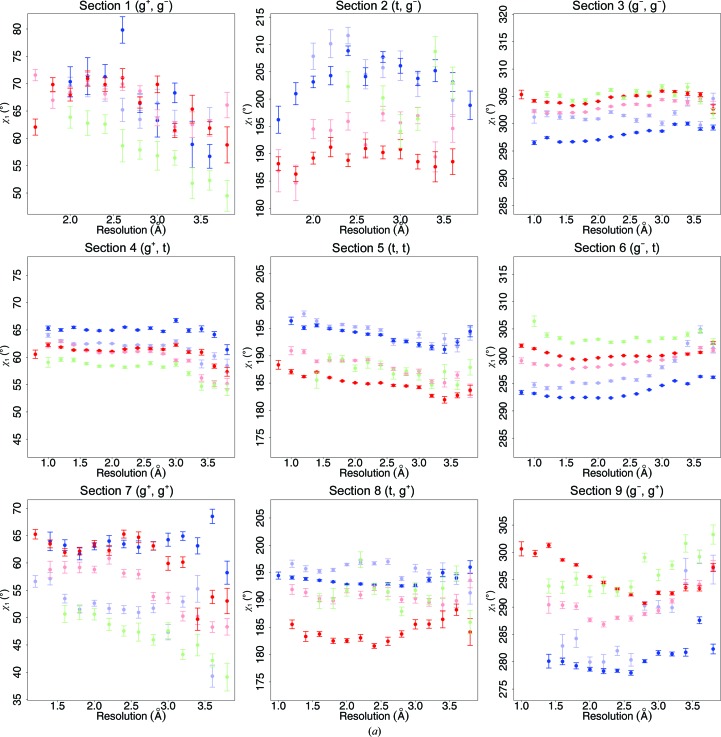

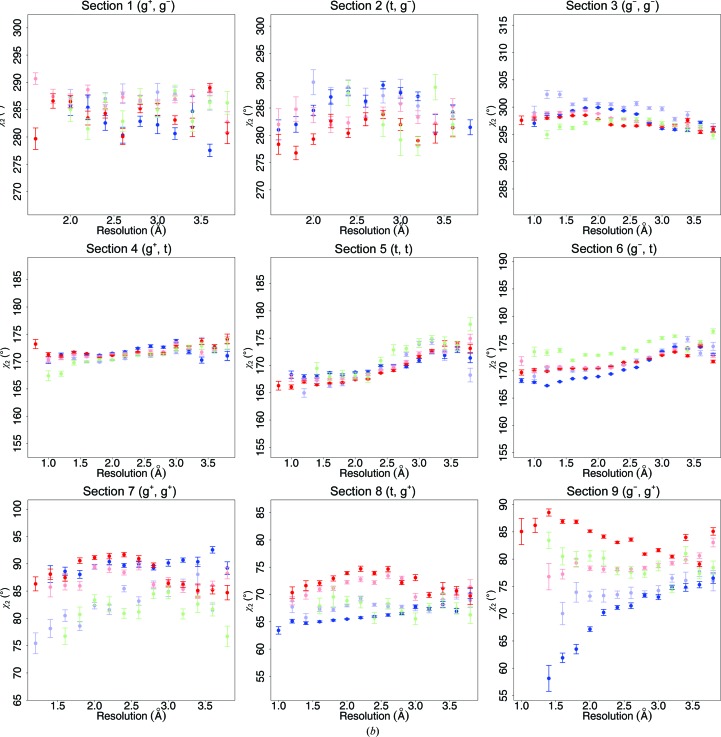
The average side-chain torsion angles of isoleucine as a function of resolution, secondary structure and backbone dihedrals. Error bars represent the standard errors of the mean. The nine panels in (*a*) show χ_1_ values. The nine panels in (*b*) show χ_2_ values. In both (*a*) and (*b*), the panels are placed in the same order as in Fig. 1 ▶. Dark blue and red are for isoleucine in an α-helix or β-strand, respectively, that have the corresponding backbone ϕ, ψ angles. The lighter coloured blue, red and green data are isoleucines that are located in a loop according to *DSSP*, but that have ϕ, ψ angles as in an α-helix, β-strand or neither, respectively. Isoleucines without missing atoms were taken from virtually every suitable X-ray structure in the PDB to obtain acceptable counting statistics. The resolution bin size was 0.2 Å, so that each dot represents the mean of at least 50 data points.

A series of studies on the dependence of the backbone angle τ on resolution, secondary structure and the refinement software used (Touw & Vriend, 2010 ▶; Jaskolski *et al.*, 2007 ▶; Karplus, 1996 ▶; Chakrabarti & Pal, 2001 ▶; Jiang *et al.*, 2010 ▶; Lundgren & Niemi, 2012 ▶) revealed the importance of using the secondary structure as determined by *DSSP* (Kabsch & Sander, 1983 ▶; Joosten, te Beek *et al.*, 2011 ▶), rather than using backbone torsion angles as a description of that secondary structure. The cooperative effects caused by the torsion-angle repeats and the repeat pattern of the hydrogen bonds cause systematic deviations of τ angles from their most relaxed value. Fig. 6 ▶ is a variant of Fig. 5 ▶. In Fig. 6 ▶ only those residues in a α-helix or β-strand are shown that have ϕ, ψ angles in agreement with the secondary structure (Touw & Vriend, 2010 ▶). The residues observed in loops according to *DSSP* (green in Figs. 2, 4 and 5) have been split in Fig. 6 ▶ into three categories, depending on whether their ϕ, ψ angles correspond to those of an α-helix, a β-strand or neither.

In most panels, the loop residues with α-helix and β-strand ϕ, ψ angles have more similar χ_1_ and χ_2_ angles than the residues actually in an α-helix and β-strand according to* DSSP*. The differences between the χ_1_ and χ_2_ angles in the three subcategories of the loop residues nevertheless tend to be significant. In Figs. 5 ▶ and 6 ▶, section 9 (χ_1_, χ_2_ = *gauche* ^−^, *gauche* ^+^) shows the most extreme resolution-dependence. This section also stands out in Fig. 1 ▶.

**Figure 7:**
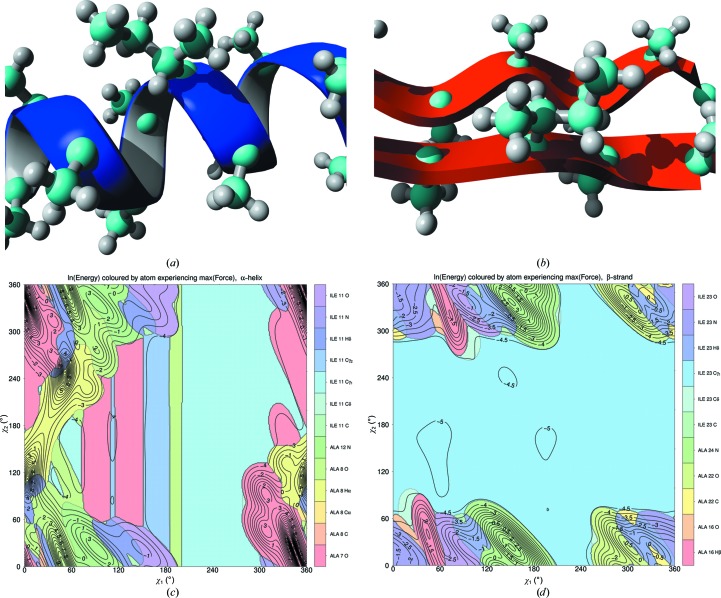

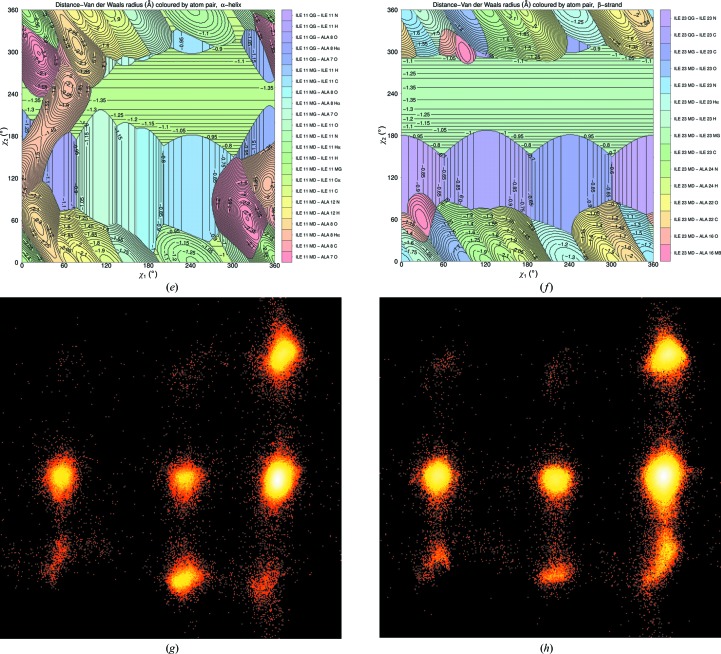
(*a*, *b*) Energy-optimized starting situation of isoleucine modelled in a polyalanine helix (*a*) (χ_1_, χ_2_ = 293, 162°) and an antiparallel polyalanine strand (*b*) (χ_1_, χ_2_ = 306, 170°). (*c*) and (*d*) show in colour for 360^2^ conformations which atom experiences the largest force using the *YASARA* NOVA force field for α-helix and β-strand, respectively. Grey lines separate coloured areas. Black lines connect conformations with equal energy. (*e*) and (*f*) show for each conformation which pair of atoms shows the largest interatomic penetration for α-helix and β-strand, respectively [1–3 interactions such as C^α^—C^γ^ or N—C^β^ are not included in (*e*) and (*f*) because they are invariable; side-chain protons are not used for clarity]. Note that different colouring schemes are used in (*c*), (*d*), (*e*) and (*f*). (*g*) and (*h*) show the frequency distribution of isoleucines in the nine sections in structures solved at better than 2.0 Å resolution for α-helix and β-strand, respectively. (*g*) and (*h*) are otherwise similar to Fig. 1 ▶.

The results shown in Figs. 1–6 are the result of a complex interplay between a large number of attractive and repulsive forces between atoms in the side chain and those in the local backbone. The fact that the isoleucines are observed in whole proteins, in which three-dimensional contacts with other residues occur, means that the plots are inevitably complicated by noise. Figs. 1–6 show that the number of local minima in the conformational space of isoleucine is limited. They also show that these minima are not typically observed with angles of 60, 180 or 300°, as could be expected from a naive interpretation of *sp* ^3^ hybridization of the atoms involved. Fig. 7 ▶ illustrates those forces that can contribute to this observation.

For two typical backbone conformations (as in Figs. 7 ▶ *a* and 7 ▶ *b*), Figs. 7 ▶(*c*) and 7 ▶(*d*) show which atom feels the largest overall force when the two side-chain torsion angles are rotated in the entire conformational space in 360 × 360 steps of 1°. In general terms, we observed that the conformations that are most populated in experimentally determined protein structures (Fig. 7 ▶ *g* and 7 ▶ *h*) correspond to areas where the energy (as computed with *YASARA* using the *YASARA* NOVA force field; Krieger *et al.*, 2002 ▶) is optimal. Neither protein structures nor force fields are perfect yet, so the computed local minima in χ_1_–χ_2_ space do not correspond perfectly with the observed maxima in the frequency plots. However, the location and depth of the local minima in the energy plots correspond well enough with the maxima in the frequency plots to explain the observed frequency differences in the 2 × 9 sections in Fig. 7 ▶. For example, section 4 is relatively less populated in α-helix than in β-strand. This is caused by the close proximity of the isoleucine C^δ^ atom to the H^α^ atom of the alanine one turn away in the α-helix. This contact does not exist in the strand situation. Repulsive forces between the isoleucine C^δ^ atom and the backbone O atom of the residue four positions earlier in the α-helix cause section 9 in the α-helix to be little populated. Section 9 in the β-strand, on the other hand, is intermediately populated. The difference in population of section 8 is fully explained by repulsive forces observed in the strand situation (with its own backbone H^α^ atom and the N and N—H of the next residue) that are absent in the helix. The top half of the banana-shaped population distribution in section 9 falls in an area that is a local energy minimum in the *YASARA* NOVA force field. The bottom half of this banana shape is situated in an area that *YASARA* NOVA considers less favourable because of repulsive interactions between the C^δ^ atom and the C, O and N atoms of the peptide plane just before the isoleucine.

**Figure 8:**
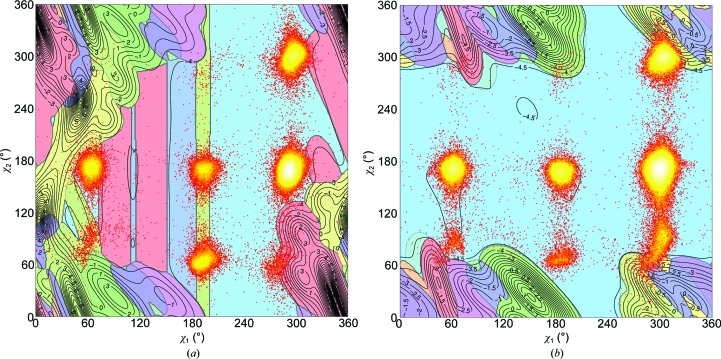
Isoleucines in an α-helix (left) or in a β-strand (right). This figure was generated by superimposing Figs. 7 ▶(*g*) and 7 ▶(*h*) on Figs. 7 ▶(*c*) and 7 ▶(*d*), respectively.

Fig. 8 ▶ shows that the observed local optima in isoleucine’s χ_1_–χ_2_ space do not agree with the optima derived from the *YASARA* NOVA (Krieger *et al.*, 2002 ▶) force field in all sections. Obviously, this disagreement does not have any quantitative value because the *YASARA* NOVA force field was not used in the X-ray refinement protocols. However, when we produce plots such as that shown in Fig. 1 ▶ for X-ray structures solved at better than 2.0 Å resolution and exclusively refined with either *CNS* (Brünger *et al.*, 1998 ▶) or *REFMAC* (Murshudov *et al.*, 2011 ▶), we find that the local optima observed in the β-strand are about 1° further away from *YASARA*’s unfavourable areas in the *CNS* structures than in the *REFMAC* structures. This is evident, for example, in sections 3 and 9, which contain the most observed isoleucine χ_1_–χ_2_ combinations in areas that *YASARA* considers unfavourable.

**Figure 9:**
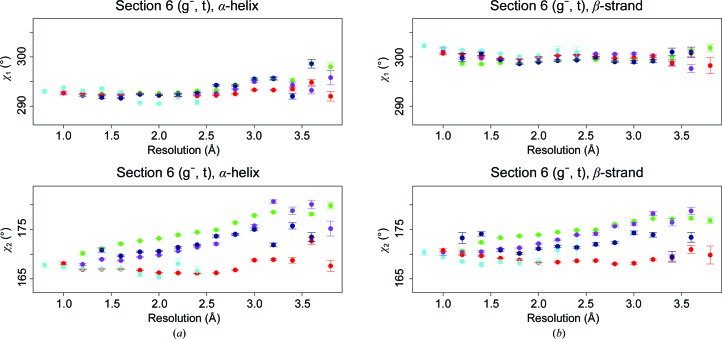
χ_1_ (top) and χ_2_ (bottom) as a function of the resolution and refinement software employed for isoleucines in an α-helix (left) and in a β-strand (right). These χ_1_ and χ_2_ values correspond to section 6, which is the most populated section in all cases (more than half of all isoleucines fall in this section). *REFMAC* (Murshudov *et al.*, 2011 ▶; red) and *SHELXL* (Sheldrick, 2008 ▶; light blue) behave similarly and so do the packages that explicitly use molecular dynamics-based force fields (*X-PLOR*, Brünger, 1992 ▶, dark blue; *CNS*, Brünger *et al.*, 1998 ▶, green). *PHENIX* (Adams *et al.*, 2010 ▶) results are in purple. Error bars represent the standard errors of the mean.

Fig. 9 ▶ shows the behaviour of χ_1_ and χ_2_ as a function of the resolution and the refinement software used for isoleucines in an α-helix in section 6 (χ_1_, χ_2_ ≃ 300, 180°). While χ_1_ shows hardly any significant differences, the χ_2_ values for *REFMAC* and *SHELXL* (Sheldrick, 2008 ▶) show systematic deviations from the values observed for the programs that use molecular dynamics-based force fields [*X-PLOR* (Brünger, 1992 ▶), *CNS* (Brünger *et al.*, 1998 ▶)] or other forms of torsion-angle restraint (*PHENIX*; Adams *et al.*, 2010 ▶).

## 4. Discussion   

Despite rapidly approaching the milestone of 100 000 entries, the PDB still does not contain sufficient data to allow us to make the detailed subset selections needed to answer some of the remaining questions. In future studies, we want to use culled data sets that contain only one copy of a particular series of similar structures that were solved by the same group using the same software. This approach was taken when selecting the data underlying Fig. 4 ▶, but the associated reduction in available data (a factor of 5–10) was unacceptably large. In many of the panels in Figs. 2, 4, 5, 6 and 9, dots are missing because we (arbitrarily) require each dot to represent at least 50 observations. A reduction in the number of PDB files by a factor of 5–10 would greatly increase the number of missing observations unless we correspondingly reduced the number of data points per dot (and thus increased the noise) in these figures.

It is practically impossible to determine how refinement programs and their sets of target values and/or force fields have changed over the years. When we split the data into two sets (structures solved before 2005 *versus* structures solved after 2004), we observe much smaller differences between the years than between secondary-structure elements. These data are available from the associated website (http://swift.cmbi.ru.nl/gv/isoleucine/).

The SCRWL rotamer library described by Dunbrack & Karplus (1993 ▶) uses the backbone torsion angles ϕ and ψ as a basis for rotamer prediction. Touw & Vriend (2010 ▶) showed that the cooperative effect of secondary-structure elements causes τ angles to be systematically different between residues in α-helix or β-strand and residues in a loop that have the same backbone torsion angles as residues in an α-helix or β-strand. Figs. 5 ▶ and 6 ▶ show that a similar effect is observed for the relationship between side-chain rotamer preference and backbone torsion angles. The larger number of interactions involved in the side-chain rotamer choices, illustrated in Figs. 7 ▶ and 8 ▶, makes this effect less straightforward to explain for the isoleucine χ_1_, χ_2_ angles than it was for τ angles.

The *YASARA* NOVA (Krieger *et al.*, 2002 ▶) force-field set is based on the original Amber (Cornell *et al.*, 1995 ▶) force-field set. Like other force-field sets such as CHARMM (MacKerell *et al.*, 1998 ▶) or GROMACS (Hess *et al.*, 2008 ▶), the Amber set is not complete. For example, it does not include terms such as induced polarization or higher order moments of aromatic planes. *YASARA*’s force-field parameters have been extensively optimized to cope with these problems by minimizing the r.m.s. difference between high-resolution X-ray structures and homology models before and after short MD simulations. Consequently, the *YASARA* force field relies less on the preconceptions of basic physics and keeps proteins closer to reality than the original Amber force field. *CNS* (Brünger *et al.*, 1998 ▶) and *X-PLOR* (Brünger, 1992 ▶) use energy force-field parameters that are somewhat similar to the values used by *YASARA*, while *PHENIX* (Adams, 2010 ▶) can use periodic torsion-angle restraints that perform a similar function. The resulting forces push the atoms in isoleucine side chains in several sections of the χ_1_, χ_2_ plot slightly away from their real position. At a resolution of around 1.0 Å this effect is negligible, because the X-ray data will decide where the atoms end up. However, at a resolution of 2 Å this effect is already significant, and at lower resolutions we observe differences of up to 10°. Side-chain torsion angle rotations of a few degrees in short side chains such as isoleucine will not lead to biologically significant errors in atomic positions. The errors enforced on the coordinates by the force field employed are nevertheless systematic in nature. It seems that refinement programs that use molecular dynamics-like energy parameters can be improved by adding more realistic, structure analysis-based, force-field terms similar to those incorporated into *YASARA* for the purpose of optimizing homology models (Krieger *et al.*, 2002 ▶).

To assist the developers of refinement software, we have determined the χ_1_, χ_2_ optima and their two-dimensional spreads for each section and each secondary-structure type in high-resolution structures. The loop residues have been split into three categories: loops with helical ϕ, ψ angles, loops with strand ϕ, ψ angles and loops with other ϕ, ψ angles. The resulting values show that it is better to use secondary-structure elements when a α-helix or β-strand is observed and ϕ, ψ angles in loops. A revised approach to torsion-angle restraints, possibly including correlated torsion-angle restraints, might need to be introduced in refinement software packages to optimally use these database-derived potentials.

## Figures and Tables

**Table 1:** Selection criteria for isoleucines

Selection parameter	Criterion
Experimental method	X-ray
Structure	Not N-terminal or C-terminal, not next to a terminus and not next to Gly, Pro or a noncanonical residue
Side-chain and backbone atoms	Not mentioned in the *WHAT_CHECK* (Hooft, Vriend *et al.*, 1996 ▶) quality report calculated with the *WHAT IF* web service (Hekkelman *et al.*, 2010 ▶)†
*B* factor, C^β^ and backbone atoms	<60.0 Å^2^ or <2.5 × the average *B* factor over C^α^
*DSSP* secondary structure	H, E or loop

† This extensive list of criteria is briefly explained at http://swift.cmbi.ru.nl/gv/isoleucine/. The criteria include, for example, atomic clashes, spurious covalent bonds, missing atoms *etc*.
